# Brain Structure Network Analysis in Patients with Obstructive Sleep Apnea

**DOI:** 10.1371/journal.pone.0139055

**Published:** 2015-09-28

**Authors:** Yun-gang Luo, Defeng Wang, Kai Liu, Jian Weng, Yuefeng Guan, Kate C. C. Chan, Winnie C. W. Chu, Lin Shi

**Affiliations:** 1 Department of Stomatology, The Second Hospital of Jilin University, Changchun, 130041, Jilin Province, China; 2 Research Center for Medical Image Computing, Department of Imaging and Interventional Radiology, The Chinese University of Hong Kong, Shatin, New Territories, Hong Kong, China; 3 CUHK Shenzhen research institute, Shenzhen, China; 4 Department of Biomedical Engineering and Shun Hing Institute of Advanced Engineering, The Chinese University of Hong Kong, Shatin, New Territories, Hong Kong, China; 5 Department of Paediatrics, The Chinese University of Hong Kong, Shatin, New Territories, Hong Kong, China; 6 Department of Medicine and Therapeutics, The Chinese University of Hong Kong, Shatin, New Territories, Hong Kong, China; National Institute for Viral Disease Control and Prevention, CDC, China, CHINA

## Abstract

Childhood obstructive sleep apnea (OSA) is a sleeping disorder commonly affecting school-aged children and is characterized by repeated episodes of blockage of the upper airway during sleep. In this study, we performed a graph theoretical analysis on the brain morphometric correlation network in 25 OSA patients (OSA group; 5 female; mean age, 10.1 ± 1.8 years) and investigated the topological alterations in global and regional properties compared with 20 healthy control individuals (CON group; 6 females; mean age, 10.4 ± 1.8 years). A structural correlation network based on regional gray matter volume was constructed respectively for each group. Our results revealed a significantly decreased mean local efficiency in the OSA group over the density range of 0.32–0.44 (*p* < 0.05). Regionally, the OSAs showed a tendency of decreased betweenness centrality in the left angular gyrus, and a tendency of decreased degree in the right lingual and inferior frontal (orbital part) gyrus (*p* < 0.005, uncorrected). We also found that the network hubs in OSA and controls were distributed differently. To the best of our knowledge, this is the first study that characterizes the brain structure network in OSA patients and invests the alteration of topological properties of gray matter volume structural network. This study may help to provide new evidence for understanding the neuropathophysiology of OSA from a topological perspective.

## Introduction

Obstructive sleep apnea (OSA) is a common sleeping disorder that is associated with neurocognitive impairment, chronically fragmented sleep and intermittent hypoxemia. Detrimental effects of OSA include daytime sleepiness, impaired work performance, increased risk of vehicular and industrial accidents, and a reduced quality of life [1]. Patients with OSA may also experience neurocognitive problems, such as deficits in memory, attention, and visuoconstructive abilities [2]. While the pathophysiology of the involvement of central nervous system during OSA is still under debate, and investigations applying neuroimaging methodologies to improve our understanding of brain alteration in individuals with OSA are needed.

Previous structural magnetic resonance imaging (sMRI) studies demonstrated that OSA patients could be found with brain structure lesions [3, 4]. Voxel-based morphometry (VBM) studies showed that OSA patients were commonly found with hippocampal atrophies [3, 4], and significant reduced gray matter (GM) volume in the caudate nucleus [5], left insular region [6], the frontal and temporo—parieto—occipital cortices [7], and cerebellar regions [3] has also been reported. Besides, after treatment, GM volume was shown to be increased in hippocampal and frontal regions [8]. Furthermore, other studies also revealed that patients with OSA showed alterations in white matter integrity and functional activation in a variety of brain areas [9–11].

In recent years, graph theoretical analysis has become increasingly popular in the neuroimaging field. Network parameters calculated based on the quantification method of small-world architecture [12] have been in widespread use to describe the topological properties of networks of different natures. For human brain, the network quantification methods provide a unique framework to test the differences in topological organization of brain network [13]. When the human brain is considered as a complex network with nodes representing brain regions containing neurons and edges representing neural connections, brain network properties can be calculated. Moreover, the brain structure networks based on morphometric measures of GM volume, cortical thickness, and surface area have been found to follow a small-world network organization as suggested by the brain networks of other connectivity modalities [14, 15]. Specifically, the structural brain network constructed using the group-level morphometric covariance (i.e., pair-wise correlation between different brain regions across a group of subjects) exhibits a good balance between segregated and integrated information processing, i.e., a small-world architecture [16]. Compared with the healthy individuals, prior research has shown alterations in topological properties of brain structural networks under disease status [17–19]. In OSA patients, the brain structural changes [20, 21] as well as the connectivity alteration of functional modalities [22, 23] was reported in previous studies, which may lead us to investigate the hypothetical alterations in brain structure correlation networks.

In the present study, we constructed the brain networks based on morphometric correlations of GM volume for OSA patients. Firstly we assumed that the brain structure network in OSA patients could follow a small-world organization, on which basis we applied graph theoretical analysis to investigate the differences in global and regional topological properties of structural brain networks between OSA patients and healthy individuals. We hypothesized that OSA may be accompanied by significant alterations in brain structure network properties, specifically, in global/regional topological properties or hub distribution. To the best of our knowledge, this is the first attempt to characterize the brain structure network in OSA patients.

## Materials and Methods

### Subjects

Twenty-five patients (OSA group; 5 female; mean age, 10.1 ± 1.8 years; age range, 7.0–13.7) with OSA and twenty normal controls (CON group; 6 females; mean age, 10.4 ± 1.8 years; age range, 7.6–13.7) were included in this study (Table 1). The study protocol was approved by the Joint Chinese University of Hong Kong—New Territories East Cluster Clinical Research Ethics Committee and informed written consents were obtained from both subjects and their parents.

**Table 1:** Demographic and neurocognitive characteristics of obstructive sleep apnea subjects (OSA) and controls (CON).

	CON (n = 20)	OSA (n = 25)	*P*-value
Age [mean(range)]	10.4 (7.6–13.7)	10.1 (7.0–13.7)	0.64
Gender (Male/Female)	14/6	20/5	0.50
OAHI [mean(range)]	0.1 (0.0–1.2)	8.1 (1.2–34.2)	< 0.001
ICV [mean(range)] (cm^3^)	1360.1 (1121.6–1594.1)	1343.0 (1123.1–1510.7)	0.65
Body height [mean(range)] (cm)^#^	133.9 (119.0–154.0)	136.1 (107.0–155.0)	0.60
Body weight [mean(range)] (kg)^#^	35.1 (22.5–59.0)	38.1 (16.6–64.7)	0.46
BMI [mean(range)] ^#^	19.1 (13.3–25.4)	20.1 (14.5–29.1)	0.47
IQ [mean(range)] ^#^	116.1 (97.0–132.0)	112.5 (87.0–140.0)	0.39
Trail making test (Part A) [mean(range)] ^#^	41.2 (24.6–63.0)	46.3 (30.0–99.0)	0.34
Trail making test (Part B) [mean(range)] ^#^	98.6 (55.0–243.0)	93.0 (61.0–160.0)	0.69
Dominant 5 rows [mean(range)] (Seconds) ^#^	70.3 (56.0–100.9)	73.6 (57.0–98.0)	0.41
Non Dominant 5 rows [mean(range)] (Seconds) ^#^	76.6 (59.0–119.0)	78.9 (28.0–113.0)	0.65
Family income (>15,000 HKD monthly) ^#^	68.8%	70.6%	> 0.999
Father’s education (Secondary or above) ^#^	88.2%	89.5%	> 0.999
Mother’s education (Secondary or above) ^#^	88.2%	94.1%	> 0.999

^#^: data with 3 missing subjects in CON group and 8 missing subjects in OSA group; OAHI = obstructive apnoea hypopnoea index; ICV = intracranial volume; BMI = body mass index; IQ = intelligence quotient; HKD = Hong Kong dollar

All the children were recruited from thirteen randomly selected primary schools from Hong Kong. A validated questionnaire [24] was finished by the parent(s) of each subject. Exclusion criteria included: 1) being ill within 4 weeks of polysomnography (PSG); 2) suffering from cardiac, renal, and neuromuscular diseases; 3) physician diagnosed attention deficit hyperactivity disorder (ADHD); 4) chromosomal abnormalities; 5) undergone upper airway surgery.

The obstructive apnoea hypopnoea index (OAHI) which was defined as the total number of obstructive apnoeas, mixed apneas, and obstructive hypopnoeas per hour of sleep was used for grouping. Because a standard diagnostic criteria for children OSA was not well established [25], we adopted a criterion of OAHI = 1.2 to define childhood OSA, which is a widely used childhood OSA definition [26]: the CON group (OAHI < 1.2 and history of snoring < 3 nights per week); OSA group (OAHI ≥ 1.2). Moreover, subjects with primary snoring (OAHI < 1.2 and history of snoring ≥ 3 nights per week) were not included in case of a potential of misclassifying with upper airway resistance syndrome (UARS). More details on PSG and definition of OSA were referred to the previous works [27, 28].

### Neurocognitive Function Assessment

In this study, the Trail Making Test (TMT) and Grooved Pegboard Test (dominant and non-dominant 5 rows), which showed high sensitivity for OSA assessment by our previous study [20], were adopted to reflect the cognitive function of subjects. TMT is a validated tool for assessment of attention, speed of processing, mental flexibility and executive functions. It contains two parts with Part A primarily emphasizing on the aspect of cognitive processing speed and part B on executive functioning (see [29] and our previous work [20] for details). The Grooved Pegboard Test which consists of a dominant hand trial and a non-dominant hand, examines the visual-fine motor coordination (see [20] for more details). In both TMT and Grooved Pegboard Test, a shorter time for finishing the test reflects a better performance. Besides, the Wechsler's Intelligence Scale for Children in Hong Kong (HK-WISC) [30] was used to assess the intelligence quotient (IQ), which is a locally validated IQ assessment for children. All these cognitive assessments were performed by an experienced psychologist.

### MRI Acquisition

All the 45 subjects were examined using a 1.5T MRI scanner (Sonata, Siemens, Erlanger, Germany) with a standard head coil. High resolution iso-voxel T1-weighted imaging (T1WI) images covering the whole brain were obtained using the magnetization prepared rapid acquisition gradient echo (MPRAGE) sequence with the following parameters: TR = 2070 ms, TE = 3.9 ms, TI = 1110 ms, flip angle = 15°, field of view = 230 × 230 mm, slice thickness = 0.9 mm (no gap), matrix = 256 × 256.

### Structural Data Processing

Statistical Parametric Mapping (SPM8, Wellcome Department of Cognitive Neurology) with the DARTEL toolbox was used to calculate the GM volume of ninety brain regions (regions of interest, ROI) in accordance with the “Automatic Anatomical Labeling” (AAL) [31]. Firstly, the image of each subject was visually inspected to exclude the cases with dramatic motion artifact, and then reoriented to approximate the Montreal Neurological Institute (MNI) space. Secondly, the brain images were segmented into GM, white matter, and cerebrospinal fluid. Then the GM images were further normalized, Jacobian modulated, and transformed to template space. To adapt the anatomical features of the pediatric brain, we used the T1WI template of the pediatric template of brain perfusion (PTBP) along with its corresponding AAL atlas, which is a population-specific template set based on the MRI data of 120 children with 7–18 years of age [32] Thirdly, an 8mm full width at half maximum (FWHM) isotropic Gaussian kernel was used to smooth the GM images. Finally, the average GM volume within each ROI of AAL was calculated and extracted using the WFU PickAtlas Toolbox implemented in SPM8.

### Correlation Matrices and Network Construction

Our correlation matrices were constructed based on pair-wise correlations between ROI pairs (negative correlations were replaced with zero). The structural correlation between a ROI pair of i and j was defined as the Pearson correlation coefficient between their mean GM volumes across the subjects within a group. The linear regression was performed in each ROI to regress out the effect of age and total brain volume. Thereafter, a correlation matrix R was established for the CON and OSA group respectively by computing the structural correlation between each pair of ROIs from all the ninety. Based on the matrix R, a matrix A was further generated by binarizing the correlation coefficient with a selected threshold. The matrix A was then considered to be an undirected graph G. The number of nodes N, two nodes of i & j, network degree K, network density D respectively represent the number of all the ROIs, a random pair of ROIs from the ninety, the number of edges, and the proportion of edges in matrix A [33].

### Threshold Selection

It should be noted that, if the same correlation coefficient level was adopted for two groups to threshold the matrices R, the resulting networks would comprise different numbers of edges, which would lead to the two networks uncomparable [34]. Therefore, we set the matrices R at a range of network densities (D_min_—D_max_), across which the network topologies of the OSA and CON group were compared. Where D_min_ was defined as the minimum density above which both of the networks were not fragmented (0.14 for this study), and D_max_ was set at 0.45 since network with more than 45% edges was not likely biological [35].

### Global Network Properties

In this study, we calculated network properties utilizing graph theory and chose graph theoretical measures to reflect the topological properties of the brain structural network [36]. The quantitative method for topological architecture of a network was used here to reflect the ability of the brain network system to communicate and organize events [12]. Firstly, the characteristic path length L was calculated as:

$$L=\frac{1}{N\left(N-1\right)}\sum_{i\neq j\in G}L_{ij}$$

In a graph G, L_ij_ was the shortest path length linking i and j. Thus L was the mean of all the shortest path lengths between two random nodes, and can be considered as a measure of network integration, i.e., the ability of the network for integrating information at global level [37]. Secondly, the clustering coefficient C was defined as the mean of the local clustering coefficients C_i_ of all the nodes:

$$C_{i}=\frac{1}{N(N_{G_{i}}-1)}\sum_{j,k\in N_{G_{i}}}1/L_{jk},\;C=\frac{1}{N}\sum_{i\in G}C_{i}$$

Here G_i_ is a subgraph of G, and the C is a measure of network segregation, reflecting the ability of a network for processing information individually and locally.

The L and C were further normalized to better summarize the topological features by comparing to 1000 matched random networks, and then represented as *λ* and *γ* [12, 38, 39].

$$\lambda =L/L_{rand},\;\gamma =C/C_{rand}$$

Then the small-worldness *σ* was then defined as:

σ=γ/λ

Moreover, the global efficiency E_glob_ and local efficiency E_loc_ were introduced. Briefly, the efficiency of a graph G E(G) was defined as the inverse of the harmonic mean of L_ij_ [33].

$$E(G)=\frac{1}{N\left(N-1\right)}\sum_{i\neq j\in G}1/L_{ij}$$

E(G) can be interpreted as E_glob_ when the whole network was considered to be the graph G. When G represented a subgraph G_i_, the local efficiency E_loc_ of the whole network can be thus defined as the average of the efficiency E(G_i_) across all subgraphs included in the whole network [33].

$$E_{loc}=\frac{1}{N}\sum_{i\in G}E(G_{i})$$

Briefly, the E_glob_ measures the efficiency of information exchange at a global level. While E_loc_ reflects the connections within the subnetworks, thus reflecting the ability of a whole network for regional information processing.

### Regional Network Properties and Hubs

Two nodal network measures of normalized betweenness centrality b_i_ and normalized degree k_i_ were applied and compared between two groups using area under the curve (AUC) method [40, 41]. The b_i_ quantified the number of shortest paths of a node, thus was a measurement the influence of a brain region over the information flow between itself and other regions. The k_i_ counted the number of edges of a node, thus measured the interaction of a region with the whole brain network.

The hubs of the network in this study were defined as the nodes with high values of normalized betweenness centrality (at least 1 standard deviation higher than the average betweenness). The hubs were considered to be the brain regions playing significant roles in information transferring and integrating for whole brain communication [17].

### Topological Metrics Comparison and Statistical Analysis

Differences in global and regional topological metrics (*λ*, *γ*, *σ*, E_glob_, E_loc_, b_i_, and k_i_) between OSA and CON groups were examined with a nonparametric permutation test [18, 42, 43] implemented in the software of Graph Analysis Toolbox developed by Hosseini et al [42]. Briefly, the metrics were calculated at a given sparsity for each group. Then the subjects in both groups were randomly assigned to either one of two groups of the same size as the original OSA and CON groups. The correlation matrices for these two newly generated groups was recalculated and binarized using the same threshold as in the real network. Finally the network metrics were calculated for each random group and their differences were compared. One thousand times of repetitions were performed to sample the permutation distributions of all metrics differences, and a two-tailed *p*-value (0.05) were calculated based on its percentile position. The regional metrics b_i_, and k_i_ were compared over the density range of 0.14–0.45 using AUC analysis. Due to the exploratory nature of this study, a less stringent threshold of *p* = 0.005 (uncorrected) was used to compensate for ninety times of comparisons.

## Results

### Demographic and Neurocognitive Information

There was no significant difference in age, gender, intracranial volume, body height, body weight, body mass index, intelligence quotient, family income, father’s education, and mother’s education between OSA and CON groups. Compared with CONs, OSA patients showed significantly higher OAHI (*p* < 0.001), as well as insignificantly longer time for finishing the TMT (Part A) and Grooved Pegboard Test (dominant and non-dominant 5 rows). Detailed statistics were summarized in Table 1.

### Global Network Properties Changes

**Fig 1:**
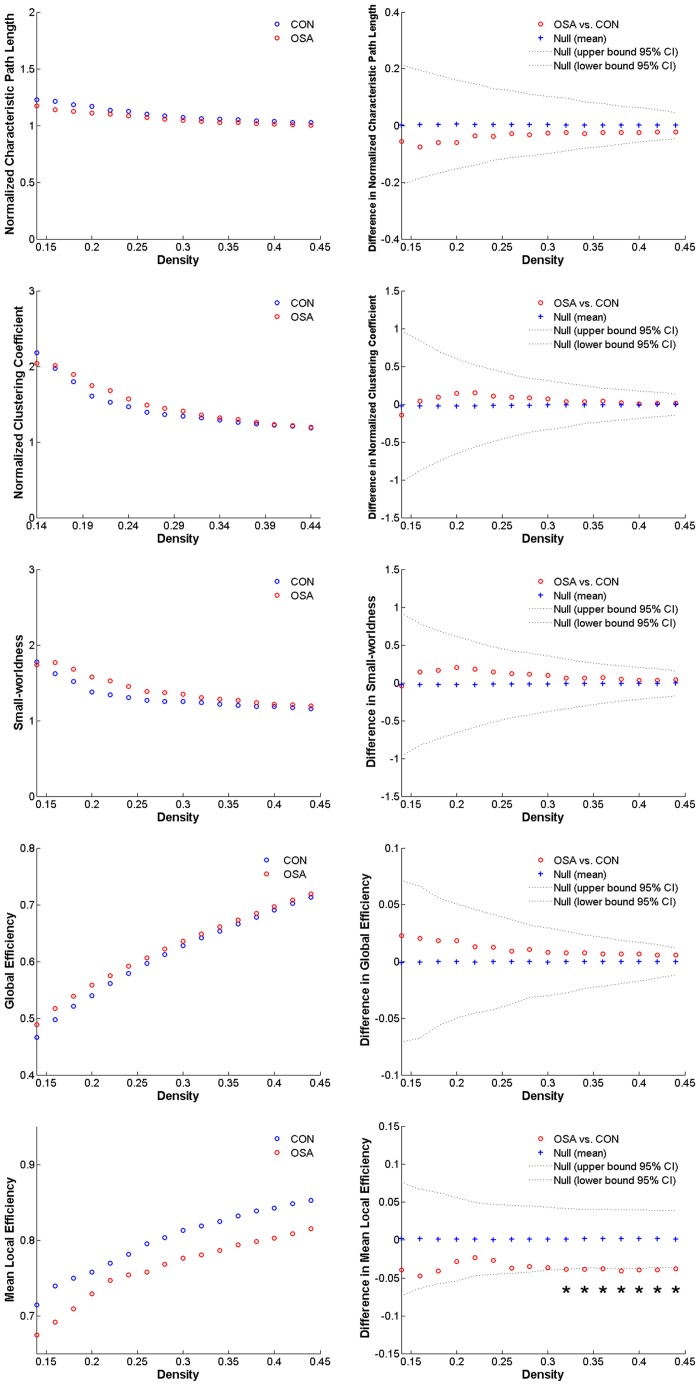
Global network properties. The normalized characteristic path length, normalized clustering coefficient, small-worldness, global efficiency, and mean local efficiency of obstructive sleep apnea patients (OSAs) and normal controls (CONs) and their between-group comparisons.

Over the density range of 0.14–0.45, both the networks of OSA and CON group exhibited efficient small-world topology (*λ* ≈ 1, *γ* >> 1, and *σ* > 1). The minimum density was determined at 0.14, above which both of the networks were not fragmented. Over the density range of 0.32–0.44, the network of the OSA group showed significantly decreased E_loc_ (Fig 1). No statistically significant difference was found in other global properties.

### Regional Network Properties Changes

**Fig 2:**
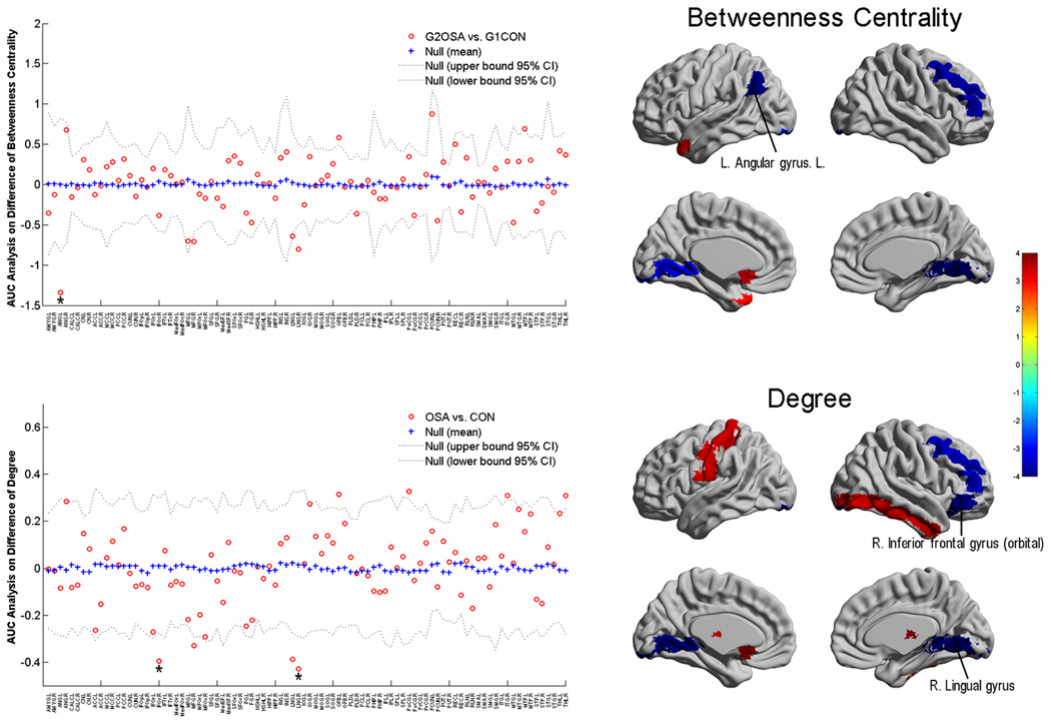
Area under the curve (AUC) analysis of between-group comparison in the regional network properties of normalized betweenness centrality and normalized degree across 0.14–0.45. The yellow-red on the brain masks (the right half of the figure) labels the regions with higher regional metric and lightblue-blue labels those with lower metric (p < 0.05, uncorrected) in obstructive sleep apnea patients (OSA) group compared with control (CON) group, with the color bar represents log (1/p-value). The brain regions with a p < 0.005 are labeled with * and illustrated with their names on the brain surfaces.

Compared with CONs, increased b_i_ of OSAs was found in the left olfactory and middle temporal pole, and decreased b_i_ in the left angular gyrus, right middle frontal gyrus, and bilateral lingual gyrus (*p* < 0.05, uncorrected). The k_i_ was increased in the right inferior occipital gyrus, left olfactory, left postcentral gyrus, right inferior temporal gyrus, and bilateral thalamus, and decreased in the right inferior frontal gyrus (orbital part), right middle frontal gyrus, and bilateral lingual gyurs (see Table 2 for detailed statistics). Among those regions, the decreased b_i_ in left angular gyrus and k_i_ in right inferior frontal and lingual gyrus were highlighted with a *p* < 0.005 (Table 2 and Fig 2).

**Table 2:** Between-group comparisons of obstructive sleep apnea (OSA) patients vs. controls (CON) in regional network properties.

Betweenness Centrality		Degree	
names	*p*-value	names	*p*-value
L. angular gyrus ↓	0.002*	R. inferior frontal gyrus (orbital part) ↓	0.004*
R. middle frontal gyrus ↓	0.024	R. middle frontal gyrus ↓	0.028
L. lingual gyrus ↓	0.042	L. lingual gyrus ↓	0.009
R. lingual gyrus ↓	0.011	R. lingual gyrus ↓	0.002*
L. olfactory ↑	0.033	R. inferior occipital gyrus ↑	0.040
L. middle temporal pole ↑	0.046	L. olfactory ↑	0.009
		L. postcentral gyrus ↑	0.031
		R. inferior temporal gyrus ↑	0.029
		L. thalamus ↑	0.034
		R. thalamus ↑	0.015

L = left, R = right; ↑/↓: increased/decreased in OSA group compared with CON group;

*: *p* < 0.005.

### Distribution of Hubs

**Fig 3:**
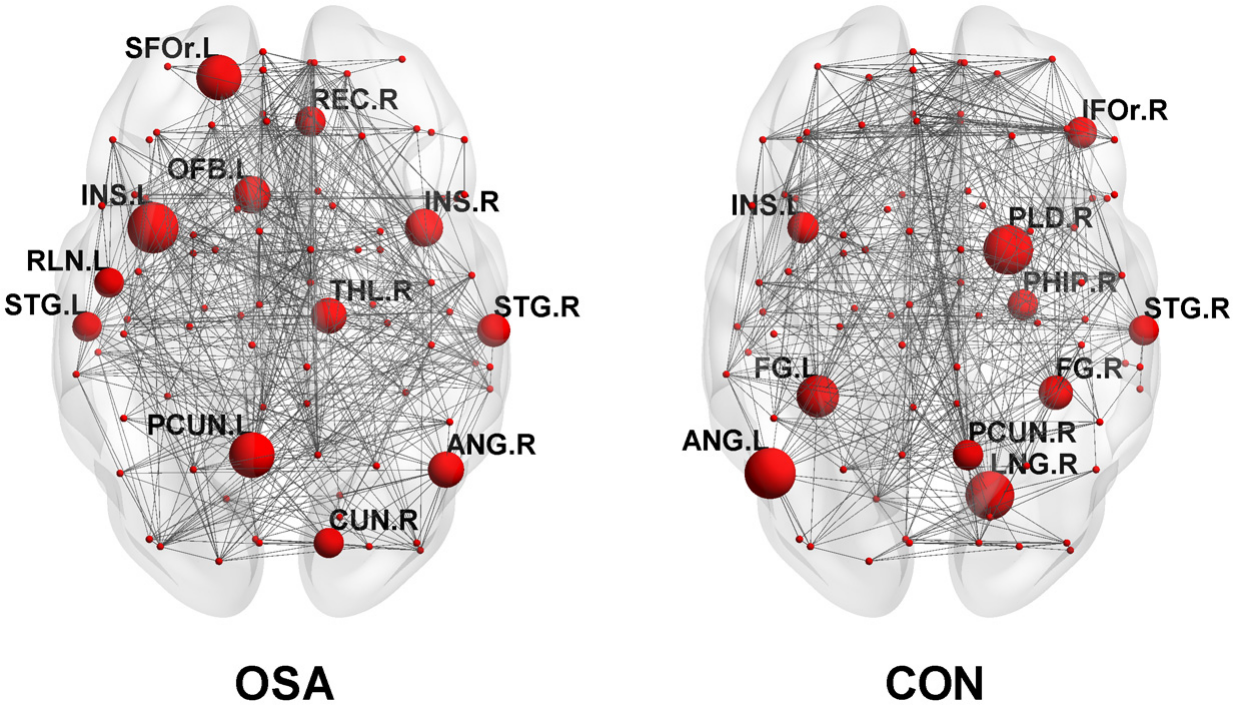
Constructed structural networks and hubs for obstructive sleep apnea (OSA) group and control (CON) group. The nodes are presented as spheres with the hubs are magnified in proportion to their b_i_ values and illustrated with the abbreviations of their names (see Table 3 for their corresponding full names).

Twelve and ten hub regions were identified respectively for OSA and CON groups (see detailed information in Table 3 and Fig 3). Among these, all the hubs were different between OSA and CON groups except that the left insular and right superior temporal gyrus were found to be present in both groups.

**Table 3:** Hub regions in the networks of obstructive sleep apnea patients (OSA) vs. controls (CON).

CON (n = 20)	OSA (n = 25)
L. angular gyrus (ANG.L)	R. angular gyrus (ANG.R)
R. inferior frontal (orbital part, IFOr.R)	R. cuneus (CUN.R)
L. fusiform (FG.L)	L. superior frontal gyrus (orbital part, SFOr.L)
R. fusiform (FG.R)	L. insular (INS.L)
L. insular (INS.L)	R. insular (INS.R)
R. lingual gyrus (LNG.R)	L. olfactory (OFB.L)
R. pallidum (PLD.R)	L. precuneus (PCUN.L)
R. Parahippocampal gyrus (PHIP.R)	R. rectus (REC.R)
R. precuneus (PCUN.R)	L. rolandic oper (RLN.L)
R. superior temporal gyrus (STG.R)	L. superior temporal gyrus (STG.L)
	R. superior temporal gyrus (STG.R)
	R. thalamus (THL.R)

L = left, R = right.

## Discussion

In our study, structural correlation network based on GM volume was constructed for each group of OSA and CON respectively, and the between-group differences in structural brain network properties were investigated. We found that the network of the OSA group suggested a significantly decreased mean local efficiency. Besides, a tendency of regional topological differences between two groups was noticed in several brain regions.

### Global Network Measures

The structural networks of both OSA patients and normal controls followed a small-word topological organization across a wide range of network densities. In line with previous studies, our results have shown a small-world organization in brain structure network of healthy individuals [14, 15].

Our results showed decreased a mean local efficiency of the brain network in the OSA group. According to previous studies, the mean local efficiency essentially quantifies how efficient the communication is between the neighbors of a node i when i is removed. Therefore, it essentially reflects the ability of fault tolerance of the network [33, 37]. Thus the current results may suggest a disrupted network architecture characterized by a status of higher vulnerability of the OSAs’ brain as well as a decreased ability of the whole network for regional information processing.

### Regional Network Measures and Network Hub Analysis

Betweenness centrality and degree combined with hub analysis were used in our study to compare the regional topological organization between two groups. Generally, though both the OSA and CON showed a small-world network, the hubs included in the two networks were largely different with only the left insular gyrus and right superior temporal gyrus being common between the two networks. The hubs of the CON network in our study were basically consistent with previous findings [36]. Thus, the currently identified differences in the hub distribution pattern perhaps underlie an altered regional topological organization of the brain network of OSA.

Specifically, some regional findings in this study may be related to the neuropathological characteristics of OSA. First, our results generally showed a more pronounced tendency of change in the right hemisphere of OSA, which may be supported by a series of previous findings of different modalities, e.g., resting-state functional MRI (rs-fMRI) [23], positron emission tomography [44], VBM [7], and surface-based techniques [45]. Secondly, a deceased betweenness centrality was noticed in the left angular gyrus, which could be in line with the previous finding that the regional homogeneity value acquired from rs-fMRI was negatively correlated with the sleep time in severe OSA patients [46]. Besides, given the angular gyrus is considered to be a component of the default-mode network, the current findings may supplementally confirm the deficit of DMN in OSA proposed by previous studies [46, 47]. Thirdly, the change in inferior frontal gyrus (IFG) which was identified to be significant in our study was highlighted in at least two previous studies [48, 49] and was attributed to the language function. However, a contradiction may be noted that a positive change was usually addressed in previous findings, e.g., an increased activation. This finding may be interesting and possibly be a characteristic of childhood OSA, since that the IGF which is closely related to language development in childhood may suggest a different alteration pattern. Fourthly, the decreased metrics of lingual gyrus also collaborated with a series findings of different modalities [50, 51], and possibly explained the deficit in spatial learning [52] and generating/recalling dreams [53, 54] in OSA.

### Limitations

There are several limitations of this study should be addressed. Firstly, the small sample size of this study may influence the confidence in the results. Thus future investigation with more subjects included is still needed. Secondly, being different from brain network of other connectivity metrics, the brain network based on structural correlation is not an individualized characteristic but a within-group description, thus the correlation between network properties and clinical measurements cannot be realized. Thirdly, the biological or functional significance of graph theoretical measures (especially the structural covariance network) may remain controversial, thus the results should be carefully interpreted.

## Conclusion

In conclusion, we have investigated the brain structure network in OSA patients, which is found to follow the small-world organization while suggests a decrease in its mean local efficiency. Besides, the OSA’s network showed a tendency of decreased regional properties in the left angular gyrus, the right lingual gyrus, and the inferior frontal gyrus. Moreover, the difference of network hub distribution also indicates an tendency of alteration in regional topological network organization in OSA. Our results may motivate future studies that investigate the underlying mechanisms of OSA.

## Supporting Information

S1 Data Raw regional grey matter volume measurement from voxel-based morphometry. This table contents the original data of age, intracranial volume, and measured grey matter volume of 90 brain regions for each subject.
(XLS)

## References

[1] Bennett LS, Barbour C, Langford B, Stradling JR, Davies RJ. Health status in obstructive sleep apnea: relationship with sleep fragmentation and daytine sleepiness, and effects of continuous positive airway pressure treatment. Am J Respir Crit Care Med. 1999; 159: 1884–1890. 10351935 10.1164/ajrccm.159.6.9808107

[2] Decary A, Rouleau I, Montplaisir J. Cognitive deficits associated with sleep apnea syndrome: a proposed neuropsychological test battery. Sleep. 2000; 23: 369–381. 10811381

[3] Macey PM, Henderson LA, Macey KE, Alger JR, Frysinger RC, Woo MA, et al Brain morphology associated with obstructive sleep apnea. Am J Respir Crit Care Med. 2002; 166: 1382–1387. 12421746 10.1164/rccm.200201-050OC

[4] Gale SD, Hopkins RO. Effects of hypoxia on the brain: neuroimaging and neuropsychological findings following carbon monoxide poisoning and obstructive sleep apnea. J Int Neuropsychol Soc. 2004; 10: 60–71. 14751008 10.1017/S1355617704101082

[5] Joo EY, Tae WS, Lee MJ, Kang JW, Park HS, Lee JY, et al Reduced brain gray matter concentration in patients with obstructive sleep apnea syndrome. Sleep. 2010; 33: 235–241. 20175407 10.1093/sleep/33.2.235 PMC2817910

[6] O'Donoghue FJ, Briellmann RS, Rochford PD, Abbott DF, Pell GS, Chan CH, et al Cerebral structural changes in severe obstructive sleep apnea. Am J Respir Crit Care Med. 2005; 171: 1185–1190. 15699018 10.1164/rccm.200406-738OC

[7] Yaouhi K, Bertran F, Clochon P, Mezenge F, Denise P, Foret J, et al A combined neuropsychological and brain imaging study of obstructive sleep apnea. J Sleep Res. 2009; 18: 36–48. 10.1111/j.1365-2869.2008.00705.x 19250174

[8] Canessa N, Castronovo V, Cappa SF, Aloia MS, Marelli S, Falini A, et al Obstructive sleep apnea: brain structural changes and neurocognitive function before and after treatment. Am J Respir Crit Care Med. 2011; 183: 1419–1426. 10.1164/rccm.201005-0693OC 21037021

[9] Macey PM, Kumar R, Woo MA, Valladares EM, Yan-Go FL, Harper RM. Brain structural changes in obstructive sleep apnea. Sleep. 2008; 31: 967–977. 18652092 PMC2491498

[10] Thomas RJ, Rosen BR, Stern CE, Weiss JW, Kwong KK. Functional imaging of working memory in obstructive sleep-disordered breathing. J Appl Physiol (1985). 2005; 98: 2226–2234. 15677733 10.1152/japplphysiol.01225.2004

[11] Sweet LH, Jerskey BA, Aloia MS. Default network response to a working memory challenge after withdrawal of continuous positive airway pressure treatment for obstructive sleep apnea. Brain Imaging Behav. 2010; 4: 155–163. 10.1007/s11682-010-9095-y 20502992

[12] Watts DJ, Strogatz SH. Collective dynamics of 'small-world' networks. Nature. 1998; 393: 440–442. 9623998 10.1038/30918

[13] Sporns O. The human connectome: a complex network. Ann N Y Acad Sci. 2011; 1224: 109–125. 10.1111/j.1749-6632.2010.05888.x 21251014

[14] He Y, Evans A. Graph theoretical modeling of brain connectivity. Curr Opin Neurol. 2010; 23: 341–350. 10.1097/WCO.0b013e32833aa567 20581686

[15] Fan Y, Shi F, Smith JK, Lin W, Gilmore JH, Shen D. Brain anatomical networks in early human brain development. Neuroimage. 2011; 54: 1862–1871. 10.1016/j.neuroimage.2010.07.025 20650319 PMC3023885

[16] Dominguez D, Gonzalez M, Serrano E, Rodriguez FB. Structured information in small-world neural networks. Phys Rev E Stat Nonlin Soft Matter Phys. 2009; 79: 21909. 10.1103/PhysRevE.79.021909 19391780

[17] Bassett DS, Bullmore E, Verchinski BA, Mattay VS, Weinberger DR, Meyer-Lindenberg A. Hierarchical organization of human cortical networks in health and schizophrenia. J Neurosci. 2008; 28: 9239–9248. 10.1523/JNEUROSCI.1929-08.2008 18784304 PMC2878961

[18] He Y, Chen Z, Evans A. Structural insights into aberrant topological patterns of large-scale cortical networks in Alzheimer's disease. J Neurosci. 2008; 28: 4756–4766. 10.1523/JNEUROSCI.0141-08.2008 18448652 PMC6670444

[19] Liu K, Shi L, Chen F, Waye MM, Lim CK, Cheng PW, et al Altered topological organization of brain structural network in Chinese children with developmental dyslexia. Neurosci Lett. 2015; 589: 169–175. 10.1016/j.neulet.2015.01.037 25597882

[20] Chan KC, Shi L, So HK, Wang D, Liew AW, Rasalkar DD, et al Neurocognitive dysfunction and grey matter density deficit in children with obstructive sleep apnoea. Sleep Med. 2014; 15: 1055–1061. 10.1016/j.sleep.2014.04.011 25023925

[21] Simmons MS, Clark GT. The potentially harmful medical consequences of untreated sleep-disordered breathing: the evidence supporting brain damage. J Am Dent Assoc. 2009; 140: 536–542. 19411520 10.14219/jada.archive.2009.0221

[22] Zhang Q, Wang D, Qin W, Li Q, Chen B, Zhang Y, et al Altered resting-state brain activity in obstructive sleep apnea. Sleep. 2013; 36: 651–659. 10.5665/sleep.2620 23633747 PMC3624819

[23] Santarnecchi E, Sicilia I, Richiardi J, Vatti G, Polizzotto NR, Marino D, et al Altered cortical and subcortical local coherence in obstructive sleep apnea: a functional magnetic resonance imaging study. J Sleep Res. 2013; 22: 337–347. 10.1111/jsr.12006 23171248

[24] Li AM, Cheung A, Chan D, Wong E, Ho C, Lau J, et al Validation of a questionnaire instrument for prediction of obstructive sleep apnea in Hong Kong Chinese children. Pediatr Pulmonol. 2006; 41: 1153–1160. 17054110 10.1002/ppul.20505

[25] Marcus CL. Childhood obstructive sleep apnea syndrome: unanswered questions. Chest. 2008; 134: 1114–1115. 10.1378/chest.08-2011 19059953

[26] Carroll JL. Obstructive sleep-disordered breathing in children: new controversies, new directions. Clin Chest Med. 2003; 24: 261–282. 12800783 10.1016/s0272-5231(03)00024-8

[27] Li AM, So HK, Au CT, Ho C, Lau J, Ng SK, et al Epidemiology of obstructive sleep apnoea syndrome in Chinese children: a two-phase community study. Thorax. 2010; 65: 991–997. 10.1136/thx.2010.134858 20965935

[28] Li AM, Au CT, Sung RY, Ho C, Ng PC, Fok TF, et al Ambulatory blood pressure in children with obstructive sleep apnoea: a community based study. Thorax. 2008; 63: 803–809. 10.1136/thx.2007.091132 18388205

[29] Tombaugh TN. Trail Making Test A and B: normative data stratified by age and education. Arch Clin Neuropsychol. 2004; 19: 203–214. 15010086 10.1016/S0887-6177(03)00039-8

[30] Psychological Corporation. Hong Kong Wechsler intelligence scale for children manual. New York, N.Y., U.S.A., 1981.

[31] Tzourio-Mazoyer N, Landeau B, Papathanassiou D, Crivello F, Etard O, Delcroix N, et al Automated anatomical labeling of activations in SPM using a macroscopic anatomical parcellation of the MNI MRI single-subject brain. Neuroimage. 2002; 15: 273–289. 11771995 10.1006/nimg.2001.0978

[32] Avants BB, Duda JT, Kilroy E, Krasileva K, Jann K, Kandel BT, et al The pediatric template of brain perfusion. Sci Data. 2015; 2: 150003 10.1038/sdata.2015.3 25977810 PMC4413243

[33] Latora V, Marchiori M. Efficient behavior of small-world networks. Phys Rev Lett. 2001; 87: 198701 11690461 10.1103/PhysRevLett.87.198701

[34] van Wijk BC, Stam CJ, Daffertshofer A. Comparing brain networks of different size and connectivity density using graph theory. PLoS One. 2010; 5: e13701 10.1371/journal.pone.0013701 21060892 PMC2965659

[35] Kaiser M, Hilgetag CC. Nonoptimal component placement, but short processing paths, due to long-distance projections in neural systems. PLoS Comput Biol. 2006; 2: e95 16848638 10.1371/journal.pcbi.0020095 PMC1513269

[36] He Y, Chen ZJ, Evans AC. Small-world anatomical networks in the human brain revealed by cortical thickness from MRI. Cereb Cortex. 2007; 17: 2407–2419. 17204824 10.1093/cercor/bhl149

[37] Rubinov M, Sporns O. Complex network measures of brain connectivity: uses and interpretations. Neuroimage. 2010; 52: 1059–1069. 10.1016/j.neuroimage.2009.10.003 19819337

[38] Maslov S, Sneppen K. Specificity and stability in topology of protein networks. Science. 2002; 296: 910–913. 11988575 10.1126/science.1065103

[39] Bassett DS, Bullmore E. Small-world brain networks. Neuroscientist. 2006; 12: 512–523. 17079517 10.1177/1073858406293182

[40] Bernhardt BC, Chen Z, He Y, Evans AC, Bernasconi N. Graph-theoretical analysis reveals disrupted small-world organization of cortical thickness correlation networks in temporal lobe epilepsy. Cereb Cortex. 2011; 21: 2147–2157. 10.1093/cercor/bhq291 21330467

[41] Ginestet CE, Nichols TE, Bullmore ET, Simmons A. Brain network analysis: separating cost from topology using cost-integration. PLoS One. 2011; 6: e21570 10.1371/journal.pone.0021570 21829437 PMC3145634

[42] Hosseini SM, Hoeft F, Kesler SR. GAT: a graph-theoretical analysis toolbox for analyzing between-group differences in large-scale structural and functional brain networks. PLoS One. 2012; 7: e40709 10.1371/journal.pone.0040709 22808240 PMC3396592

[43] Bullmore ET, Suckling J, Overmeyer S, Rabe-Hesketh S, Taylor E, Brammer MJ. Global, voxel, and cluster tests, by theory and permutation, for a difference between two groups of structural MR images of the brain. IEEE Trans Med Imaging. 1999; 18: 32–42. 10193695 10.1109/42.750253

[44] Antczak J, Popp R, Hajak G, Zulley J, Marienhagen J, Geisler P. Positron emission tomography findings in obstructive sleep apnea patients with residual sleepiness treated with continuous positive airway pressure. J Physiol Pharmacol. 2007; 58 Suppl 5: 25–35. 18204112

[45] Torelli F, Moscufo N, Garreffa G, Placidi F, Romigi A, Zannino S, et al Cognitive profile and brain morphological changes in obstructive sleep apnea. Neuroimage. 2011; 54: 787–793. 10.1016/j.neuroimage.2010.09.065 20888921 PMC4169712

[46] Peng DC, Dai XJ, Gong HH, Li HJ, Nie X, Zhang W. Altered intrinsic regional brain activity in male patients with severe obstructive sleep apnea: a resting-state functional magnetic resonance imaging study. Neuropsychiatr Dis Treat. 2014; 10: 1819–1826. 10.2147/NDT.S67805 25278755 PMC4179755

[47] Prilipko O, Huynh N, Schwartz S, Tantrakul V, Kim JH, Peralta AR, et al Task positive and default mode networks during a parametric working memory task in obstructive sleep apnea patients and healthy controls. Sleep. 2011; 34: 293–301. 21358846 10.1093/sleep/34.3.293 PMC3041705

[48] Ayalon L, Ancoli-Israel S, Klemfuss Z, Shalauta MD, Drummond SP. Increased brain activation during verbal learning in obstructive sleep apnea. Neuroimage. 2006; 31: 1817–1825. 16626972 10.1016/j.neuroimage.2006.02.042

[49] Castronovo V, Canessa N, Strambi LF, Aloia MS, Consonni M, Marelli S, et al Brain activation changes before and after PAP treatment in obstructive sleep apnea. Sleep. 2009; 32: 1161–1172. 19750921 10.1093/sleep/32.9.1161 PMC2737574

[50] Joo EY, Tae WS, Han SJ, Cho JW, Hong SB. Reduced cerebral blood flow during wakefulness in obstructive sleep apnea-hypopnea syndrome. Sleep. 2007; 30: 1515–1520. 18041484 10.1093/sleep/30.11.1515 PMC2082095

[51] Huynh NT, Prilipko O, Kushida CA, Guilleminault C. Volumetric Brain Morphometry Changes in Patients with Obstructive Sleep Apnea Syndrome: Effects of CPAP Treatment and Literature Review. Front Neurol. 2014; 5: 58 10.3389/fneur.2014.00058 24808886 PMC4010762

[52] Tsang CS, Chong SL, Ho CK, Li MF. Moderate to severe obstructive sleep apnoea patients is associated with a higher incidence of visual field defect. Eye (Lond). 2006; 20: 38–42. 15650758 10.1038/sj.eye.6701785

[53] Pagel JF. Non-dreamers. Sleep Med. 2003; 4: 235–241. 14592328 10.1016/s1389-9457(02)00255-1

[54] Bischof M, Bassetti CL. Total dream loss: a distinct neuropsychological dysfunction after bilateral PCA stroke. Ann Neurol. 2004; 56: 583–586. 15389890 10.1002/ana.20246

